# Ultrastable Polypyrrole Stabilized by Hyper-Cross-Linked Poly(styrene-*co*-divinylbenzene) for Long-Cycle Supercapacitor Applications

**DOI:** 10.1021/acsapm.5c03208

**Published:** 2025-11-04

**Authors:** Petr Šálek, Manoj Karakoti, Sonal Gupta, Libor Kobera, Adriana Šturcová, Miloš Steinhart, Jiří Brus, Islam M. Minisy, Stefan Breitenbach, Christoph Unterweger, Jiřina Hromádková, Patrycja Bober

**Affiliations:** † Institute of Macromolecular Chemistry, Czech Academy of Sciences, 162 00 Prague, Czech Republic; ‡ 273939 Wood K plus − Kompetenzzentrum Holz GmbH, 4040 Linz, Austria

**Keywords:** composite, electrochemical stability, hyper-cross-linked poly(styrene-*co*-divinylbenzene), polypyrrole, supercapacitor

## Abstract

Polypyrrole (PPy) is a promising conducting polymer for supercapacitor electrodes, but its structural degradation during repeated cycling limits its long-term stability. Here, we report the design of a composite based on PPy chemically stabilized by hyper-cross-linked poly­(styrene-*co*-divinylbenzene) (HPStDVB) microparticles (PPy/HPStDVB) working as a scaffold. This composite possesses a more compact structure, preventing interruptions in the conjugation of PPy. As a result, the PPy/HPStDVB electrode exhibits remarkable electrochemical durability, maintaining its capacitance over 20,000 charge–discharge cycles and demonstrating a dominant capacitive contribution (77.3%) in the hybrid supercapacitor cell. These more stable electrochemical properties of PPy on HPStDVB arise from the interactions of the HPStDVB backbone with PPy, resulting in a more stable structure due to less dynamic functional groups. This study introduces a simple yet efficient strategy for mechanically reinforced conducting-polymer electrodes, providing a new route toward durable, high-performance supercapacitors.

## Graphical abstract

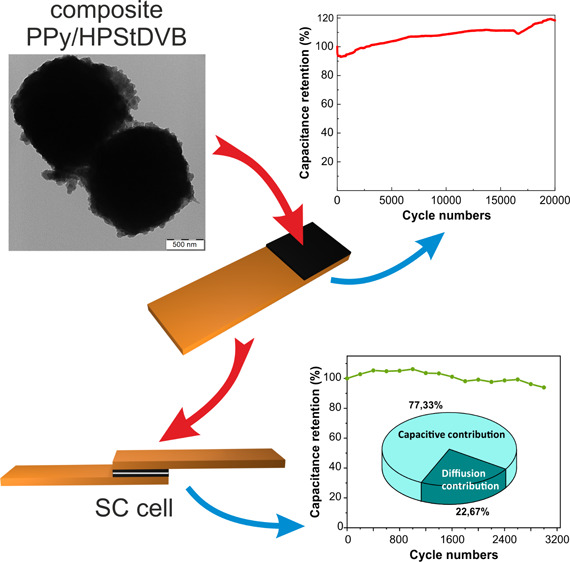

## 1 Introduction

Nowadays, society creates high energy demands that have a massive impact on the global climate and environment. Thus, renewable and sustainable energy sources are key technologies which can satisfy current energy needs and alleviate concerns with energy storage, sufficient power density, reusability, longevity, and lightweight.^1^ Significant attention has been dedicated to supercapacitors (SCs), representing a promising and effective solution for energy storage and supply requirements. SCs are classified as energy storage systems that store electrical energy on the basis of charge separation utilizing two electrodes and a separator to store and deliver electric energy. According to the mechanism of energy storage, SCs can be divided into electric double-layer capacitors (linear and voltage-independent capacitance, energy storage and release by nonfaradaic adsorption/desorption) and pseudocapacitors (energy storage by reversible faradaic redox reactions).^2,3^ SCs have improved properties over traditional batteries and capacitors including faster charging and discharging times, longer cycle life, higher power density, and designed structure and composition.^4^ Thus, SCs have been applied in the fields of microscale devices, electronics, transportation, and energy harvesting.^5^ Due to high conductivity, high porosity, and large surface area, carbon-based materials, covering activated carbon, graphene, carbon nanotubes, biomass-derived porous carbon, and mesoporous carbon, have been widely employed as electrode materials for the construction of electric double-layer capacitors.^6^ Meanwhile, transition metal oxides/hydroxides, transition metal sulfides/selenides, conducting polymers, and nanocarbon materials with oxygen-/nitrogen-reactive groups represent typical materials for the construction of pseudocapacitors.^5^

Polypyrrole (PPy) and its derivatives are common and well-known conductive polymers with large specific capacitance, a broad range of electrical conductivity, exceptional chemical stability, and low cost.^7^ Moreover, characteristics of nanostructured PPy, such as excellent electrical conductivity, high carrier mobility, improved electrochemical activity and optical properties, and larger surface area, are superior to bulk PPy and make it advantageous for application in pseudocapacitive electrode materials. However, the main disadvantages of PPy-based SCs lie in reduced cycle life and stability due to exposure to continuous cycling, leading to undesired significant volume changes, and in low achieved specific capacitance due to a reduced exposed surface for contact with an electrolyte.^8,9^ Despite this, several PPy composite materials have been designed to overcome these limitations. For instance, the synergic effect of PPy nanocomposites with metal oxides has been proven as a very effective solution for improving electron transfer ability, capacitance, and cycling stability.^10–14^ Tuning of electrochemical and structural properties can also be achieved by PPy preparation with various oxidizing agents (e.g., ferric chloride, ammonium peroxydisulfate, and hydrogen peroxide) or in the presence of cationic dyes that can further be combined with anionic surfactants. This strategy enables the preparation of PPy nanowires, nanorods, and nanotubes with improved electrochemical performance.^15,16^ Another approach for/to overcoming the limitations is the optimization of polymerization conditions for the preparation of PPy.^17^ Despite the great advantages of pseudocapacitive materials, they also have some disadvantages over electronic double-layer capacitors. Nevertheless, the combination of these two types of materials results in a class of SCs known as hybrid SCs. Typically, PPy combined with carbon-based materials, such as graphene and graphene oxide, exhibits superior stability, high electrical conductivity, high performance, and low cost.^18^ Hybrid SCs are mostly made by drop-casting using an extra binder and by electrodeposition methods.^8^ As mentioned above, however, the most serious issue with PPy-based SCs lies in undesired significant volume changes during charge/discharge cycles, and it may be overcome with an additional structural component supporting PPy mechanical properties.

Hyper-cross-linked materials represent a unique class of materials with post-cross-linked structures of enhanced rigidity, micro/mesoporosity, high specific surface area, and sorption properties. Such polymers have found applications in diverse fields including environmental, separation, and biomedical sciences, as well as electronics, among others, due to their unique combination of properties arising from their highly cross-linked and porous structures.^19^ Poly­(styrene) (PSt) and poly­(styrene-*co*-divinylbenzene) (PStDVB) are the most studied polymers for manufacturing hyper-cross-linked polymers. Briefly, PSt chains in solution, or highly solvated PStDVB with a thermodynamically good solvent, are chloromethylated and then hyper-cross-linked by the addition of a Lewis acid as a catalyst, which results in the formation of methylene bridges between polymer chains and additional micro- and mesoporous structures.^20^ Hyper-cross-linked materials are not only restricted to hyper-cross-linked PSt (HPSt) and hyper-cross-linked PStDVB (HPStDVB), there are also reports about different strategies for the preparation of hyper-cross-linked poly­(vinylbenzyl chloride-*co*-divinylbenzene)­s, polysulfones, polyacrylates, polyanilines, polypyrrole, polyacetylenes, and substituted pyridines.^21,22^ In 2019, Cai et al. prepared 130 nm PStDVB spheres by emulsion polymerization, which were further hyper-cross-linked with an AlCl_3_ catalyst, coated with PPy, carbonized, and finally KOH-activated with the aim of developing a novel core–shell composite system for CO_2_ storage.^23^ Since hyper-cross-linked polymers have a high cross-linking degree and a more rigid network, we hypothesized that such a polymer material could serve as a solid support for PPy, promoting and improving chemical stability by reducing segmental dynamics, resulting in prolonged electrochemical stability. However, up to now, no work has been published about the development of a similar PPy/HPSTDVB system as a material for SCs.

Herein, we report an innovative route for the fabrication of a PPy/HPStDVB SC with enhanced electrochemical stability via pyrrole surface polymerization on HPStDVB microparticles. Initial 1.80 μm PStDVB microparticles were prepared by dispersion polymerization and subsequently chloromethylated and hyper-cross-linked. The resulting HPStDVB microparticles with slightly reduced size were employed as a solid support during pyrrole polymerization. The final PPy/HPStDVB SC was composed of ∼30 nm PPy nanoparticles coated on HPStDVB microparticles, with increased *S*_BET_ (20%) and total pore volume (25%) in comparison with HPStDVB. Such a novel material demonstrates excellent electrochemical cycle stability and maintains its capacitance over 20,000 cycles.

## 2 Experimental Section

### 2.1 Materials

Styrene (St) and divinylbenzene (DVB; 64% m- and 36% p-isomers) from Synthos (Kralupy nad Vltavou, Czech Republic) were vacuum-distilled. Ethanol (EtOH) for UV spectroscopy and 1,2-dichlorethane (DCE) were purchased from Lach-Ner (Neratovice, Czech Republic). 2,2′-Azobis­(2-methylpropionitrile) (AIBN; crystallized from ethanol), chloromethyl methyl ether (CMME), anhydrous iron­(III) chloride, polyvinylpyrrolidone K30 (PVP), and Nafion 117 solution (∼5% in a mixture of lower aliphatic alcohols and water) were purchased from Sigma-Aldrich (St. Louis, MO, USA). Pyrrole (≥98%; used as received without any further purification) from Sigma-Aldrich (China) and iron­(III) chloride hexahydrate from Sigma-Aldrich (Germany) were obtained.

### 2.2 Preparation of Poly­(styrene-*co*-divinylbenzene) (PStDVB) by Dispersion Polymerization

PStDVB microparticles were prepared according to a modified procedure by Zhang et al.^24^ Dispersion polymerization of St with DVB was conducted in a glass reaction vessel (100 mL) equipped with an anchor-type stirrer. First, PVP (1.04 g; 1.7 wt % relative to the medium) was dissolved in a mixture of ethanol (55 g) and distilled water (6.188 g). Second, AIBN (0.1375 g; 4 wt % relative to St) was dissolved in a mixture of St (3.438 g) and DVB (0.1719 g; 5 wt % relative to St). Both solutions were mixed, charged in the reactor, and purged with nitrogen for 15 min. Finally, the polymerization was allowed to proceed at 74 °C for 24 h under stirring (100 rpm). At the end of polymerization, the PStDVB particles were removed by centrifugation (8000 rpm), washed with ethanol (40 mL) ten times, and freeze-dried for 24 h.

### 2.3 Preparation of Hyper-Cross-Linked PStDVB Microparticles (HPStDVB)

PStDVB microparticles were chloromethylated and hyper-cross-linked according to a previously reported procedure.^25^ Briefly, the PStDVB microparticles (3 g) were swollen in anhydrous DCE (48 mL) at room temperature for 24 h in a 100 mL round-bottomed flask. The mixture was cooled to −15 °C in an ice/sodium chloride bath under magnetic stirring; CMME was added (2.19 mL), and the mixture was chloromethylated at this temperature for 1 h. Then, anhydrous iron­(III) chloride (2.33 g) was added, and the mixture was refluxed at 80 °C for 20 h. After completion of the reaction, the HPStDVB particles were kept in anhydrous DCE (200 mL) for 12 h under magnetic stirring, filtered, washed with ethanol (30 mL) ten times, and freeze-dried for 24 h.

### 2.4 Coating of HPStDVB Microparticles with Polypyrrole

HPStDVB microparticles (1 g) were coated with 20 wt % of PPy (weight was calculated with respect to the pyrrole monomer) by the polymerization of pyrrole (0.207 mL) with iron­(III) chloride hexahydrate (2.02 g) in an aqueous medium at room temperature. First, the HPS particles were soaked in 7.3 mL of deionized Milli-Q water overnight. Then, pyrrole was added, and the mixture was kept for 2 h under magnetic stirring. Finally, the iron­(III) chloride hexahydrate solution (7.5 mL) was added to the reaction mixture. The reaction mixture was thus composed of 0.2 M pyrrole and 0.5 M iron­(III) chloride (the oxidant/pyrrole mole ratio was 2.5). After 24 h, the PPy/HPStDVB composite microparticles were collected by filtration, washed with 50 mL of 0.2 M HCl and 100 mL of ethanol, and left to dry over silica gel until a constant weight was obtained.

### 2.5 Characterization

The particle size in the dry state, size distribution, and morphology were analyzed by field-emission scanning electron microscopy (SEM) and transmission electron microscopy (TEM). For SEM analyses, the samples were dispersed on carbon tape and uniformly coated with a thin platinum layer (thickness 4 nm). The coating was carried out by cathode sputtering under vacuum conditions (sputter coater SCD-050, BAL-TEC). Imaging was performed using SEM (MAIA3, TESCAN, Czech Republic). For TEM analyses, the sample was embedded in resin (London Resin White). Ultrathin sections for TEM were prepared by cutting them with a diamond knife using an ultramicrotome (Ultracut UCT, Leica) at room temperature. The ultrathin sections were collected on Cu microscope grids. Imaging was performed using a TEM microscope (Tecnai G2 Spirit Twin 12, Czech Republic). The number-average diameter (*D*_*n*_), weight-average diameter (*D*_*w*_), and dispersity (*Đ* = *D*_*w*_/*D*_*n*_) were calculated using ImageJ software (Tescan Digital Microscopy Imaging, Brno, Czech Republic) by counting at least 500 individual particles on SEM micrographs. *D*_*n*_ and *D*_*w*_ can be expressed as follows:

$$D_{n}=\sum n_{i}D_{i}/\sum n_{i}$$ 1

$$D_{w}=\sum n_{i}D_{i}^{4}/\sum n_{i}D_{i}^{3}$$ 2

where *n*_*i*_ and *D*_*i*_ are the number and diameter of the *i*-th microsphere, respectively.

The HPStDVB and PPy/HPStDVB particles were characterized by small-angle X-ray scattering (SAXS). SAXS measurements were performed by means of a point-focusing SAXS instrument (originally Molecular Metrology, USA; later considerably upgraded by SAXSLAB, now Xenocs). The CuKα beam with a wavelength of λ = 0.154 nm was generated by Rigaku Micromax-003 which is a low-power microsource equipped with X-ray optics working at *U* = 50 kV and *I* = 0.6 mA. Scattering was detected by a 2D detector (Pilatus3 300 K Dectris, with 487 × 619 pixels, each of the size 172 × 172 μm) at three different distances, 1.545, 1.135, and 0.524 m, from the sample. The sample-to-detector distance was calibrated in each position by means of a Ag Behenate standard. Data were azimuthally averaged, adjusted to absolute scale using a glassy carbon standard, and merged. The results are scattering dependencies in a reliable *q* interval from 0.05 to 5 nm^–1^, where *q* is the magnitude of the scattering vector *q = 4*π sin­(θ)/λ, and θ is half of the scattering angle which was reached. The scattering data were fitted using a structure model by means of SASfit software (Ingo Bressler).

Specific surface area and total pore volume of the materials were determined by physisorption measurements using an automatic volumetric sorption analyzer Autosorb-iQ (Anton Paar QuantaTec Inc., Graz, Austria) with nitrogen at 77 K. Prior to analysis, the samples were degassed under vacuum at 90 °C until a constant mass was achieved.

Elemental analysis was carried out to estimate C, H, N, Fe, and Cl contents using a Thermo Fisher Scientific FlashSmart Elemental Analyzer.

Solid-state NMR (ssNMR) spectra were collected by using a 700 MHz Bruker Avance Neo NMR spectrometer (*B*_0_ = 16.4 T) at a Larmor frequency ν­(^13^C) = 224.684 MHz, using a double-resonance 3.2 mm magic-angle spinning (MAS) probe. All MAS NMR experiments were recorded with a SPINAL 64 decoupling sequence. All ^13^C ssNMR experiments were performed at 17 kHz and a 2 s recycle delay. ^13^C CP/MAS NMR spectra were recorded with a 0.8 ms spin-lock at 20480 scans.^26^ The site-specific measurement of ^1^H–^13^C dipolar couplings under Lee–Goldburg (LG) conditions was achieved by using the two-dimensional (2D) phase-inverted LG recoupling under MAS (PILGRIM).^27^ The length of the polarization-inversion period was 0.5 ms, and the Lee–Goldburg cross-polarization was incremented by 22.8 μs. The experiments were performed at spinning frequency ω_*r*_/2π = 17.0 kHz, the recycle delay was 2 s, and the *t*_1_ evolution period consisted of 36 increments, each made of 1536 scans. The ^13^C chemical shift was calibrated using α-glycine (176.03 ppm; carbonyl signal) as the external standard. The samples were kept and packed into ZrO_2_ rotors under an Ar atmosphere. The NMR experiments were performed at a temperature of 303 K, and temperature calibration was performed to compensate for frictional heating of the samples.^28^ All NMR spectra were processed and simulated using the TopSpin 3.5 pl7 software package.

A Thermo Nicolet Nexus 870 FTIR spectrometer, purged with dry air and equipped with a liquid-nitrogen-cooled MCT (mercury–cadmium–telluride) detector, was used for obtaining infrared spectra. All attenuated total reflectance (ATR) FTIR spectra were acquired using a Golden Gate single-reflection ATR accessory (Specac Ltd.) equipped with a diamond internal reflection element. Spectra acquisition parameters were as follows: resolution 4 cm^–1^, 256 scans. Linear baseline corrections and ATR corrections were applied.

Electrochemical studies were carried out using a Metrohm AUTOLAB PGSTAT302N potentiostat in both three-electrode and two-electrode systems at room temperature. In the three-electrode system, a platinum wire, a Ag/AgCl electrode, and a glassy carbon (GC; diameter = 3 mm) electrode were used as the counter, reference, and working electrodes, respectively. The electrolyte used was 1 M HCl. For the electrochemical measurements, ∼9 mg of finely ground sample mixed with ∼2 mg of carbon black was dispersed in a mixture of 800 μL of isopropanol, 1.18 mL of water, and 20 μL of Nafion 117. The prepared mixture was sonicated for 1 h at room temperature.^18^ Next, 1 μL of the PPy/HPStDVB particle dispersion was drop-cast on the GC; then, the electrode was soaked in the electrolyte for 24 h. The system was purged with nitrogen before electrochemical measurement.

In addition, for the two-electrode system (or cell), the above PPy/HPStDVB particle dispersion was coated over two carbon cloth pieces in an area of 1 × 1 cm^2^. The PPy/HPStDVB-coated electrodes were kept in an oven for drying at 75 °C for 24 h. A symmetric cell was fabricated by arranging two PPy/HPStDVB-coated electrodes in a sandwich-like structure and placing 1 M HCl-soaked filter paper as the separator between them (Figure 1).

**Figure 1:**
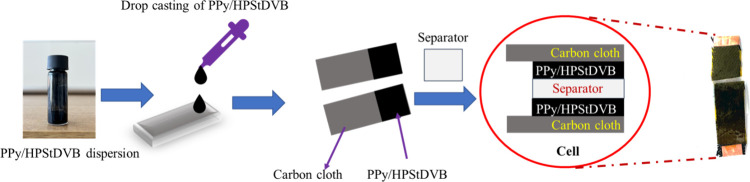
Fabrication of the PPy/HPStDVB-based SC cell.

The electrochemical performance of both systems was carried out by cyclic voltammograms (*CV*), electrochemical impedance spectroscopy (EIS), and galvanostatic charge discharge (GCD).^29^ CV was recorded at various scan rates to evaluate areal capacitance (*C*_A_) in mF/cm^2^ and gravimetric capacitance (*C*_g_) in F g^–1^ for the three-electrode system and cell, respectively, using the following equations:

$$\begin{array}{cc} C_{A}=\frac{\int_{V_{1}}^{V_{2}}IdV}{Av\Delta V} & \mathrm{for}\;\mathrm{three}-\mathrm{electrode} \end{array}$$ 3

$$\begin{array}{cc} C_{g}=\frac{\int_{V_{1}}^{V_{2}}IdV}{mv\Delta V} & \mathrm{for}\;\mathrm{cell} \end{array}$$ 4

where ∫*I*d*V* is the integral area under the *CV*, m (g) depicts the mass of drop-cast material (∼1 mg), *A* is the area of electrode (0.07 cm^2^), *v* represents the scan rate in mV/s, and Δ*V* is the potential window, ranging from 0 to 0.75 V.

EIS measurements were performed with the FRA32 M Metrohm AUTOLAB module in the frequency range of 0.1 Hz to 10^5^ Hz with an amplitude of 5 mV. *C*_A_ and *C*_g_ were calculated using the following equations:

$$\begin{array}{cc} C_{A}=\frac{1}{2A\pi fZ^{\prime \prime }} & \mathrm{for}\;\mathrm{three}-\mathrm{electrode} \end{array}$$ 5

$$\begin{array}{cc} C_{g}=\frac{1}{2m\pi fZ^{\prime \prime }} & \mathrm{for}\;\mathrm{cell} \end{array}$$ 6

where *f* is the frequency in Hz, and *Z*″ is the maximum value at the imaginary part of the impedance.

Furthermore, the charging/discharging behavior of the three-electrode system and the cell was performed at different current densities. *C*_A_ and *C*_g_ were evaluated from GCD using the following equations:

$$\begin{array}{cc} C_{A}=\frac{I\times △t}{A△V} & \mathrm{for}\;\mathrm{three}-\mathrm{electrode} \end{array}$$ 7

$$\begin{array}{cc} C_{g}=\frac{I\times △t}{m△V} & \mathrm{for}\;\mathrm{cell} \end{array}$$ 8

where *I* is the current density in ampere, △*t* is the discharging time in second, and △*V* is the potential window of GCD in volt.

Furthermore, the energy density (*E*_d_) and power density (*P*_d_) were calculated for the cell using following equations:

$$E_{d}=\frac{1}{2}C△V^{2}$$ 9

$$P_{d}=\frac{E_{d}}{△t}\times 3600$$ 10

where △*V* is the applied potential during the charging and discharging in volt, △*t* is the discharging time in second, *E*_d_ is expressed in Wh/kg, and *P*_d_ in W/kg.

## 3 Results and Discussion

Dispersion polymerization is conventionally employed for the preparation of various micron-sized particles. Here, St was copolymerized with 5 wt % DVB, resulting in PStDVB particles with *D*_*n*_ = 1.80 μm, *D*_*w*_ = 1.83, and *Đ* = 1.017 (see Supporting Information (SI), Figure S1). These PStDVB particles were almost spherical in shape, with slight surface roughness as a result of the slightly inhomogeneous cross-linking of the particles with DVB.^30^ However, the presence of H_2_O in the polymerization medium significantly suppressed the formation of completely irregular, aggregated, and broadly sized particles as it is typical for the dispersion polymerization of St with high DVB concentrations (≥1 wt %).^24^ Then, 1.80 μm PStDVB microparticles were chloromethylated with CMME and hyper-cross-linked via Friedel–Crafts alkylation to introduce a porous structure into nonporous PStDVB microparticles. After hyper-cross-linking, the diameter of HPStDVB microparticles was reduced to *D*_*n*_ = 1.64 μm (Figure 2a) compared to the starting PStDVB microparticles (Figure S1), due to the formation of a densely cross-linked network inside the HPStDVB microparticles and their shrinkage. Moreover, Figure 2a and c illustrates that HPStDVB microparticles were partially disrupted by the hyper-cross-linking reaction, and small pores were successfully introduced into the HPStDVB microparticles. This observation was also confirmed by a TEM image of the cross-section of HPStDVB microparticles, which documents the presence of fine ∼6 nm pores inside the HPStDVB microparticle (see SI, Figure S2).

**Figure 2:**
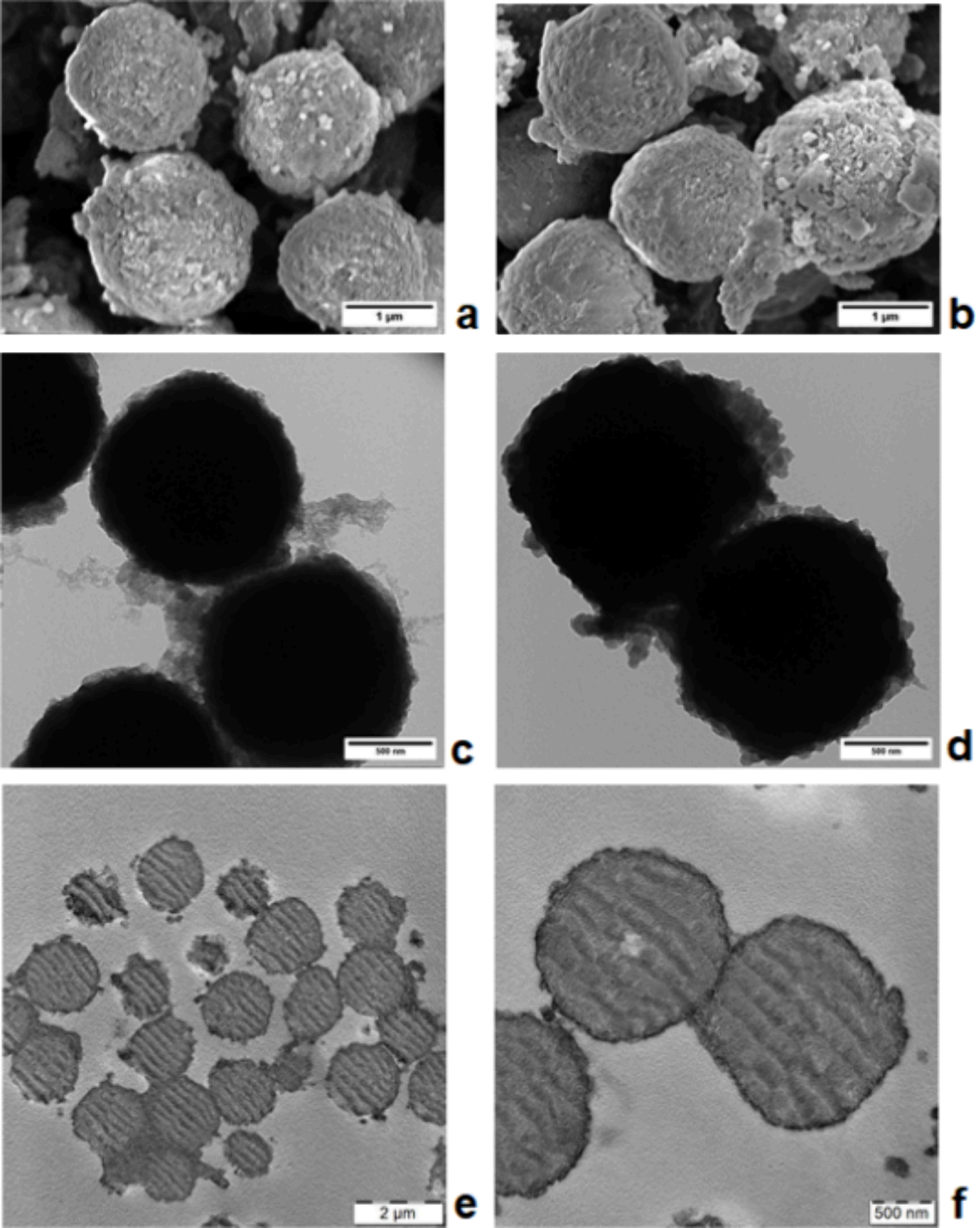
SEM micrographs and TEM images of (a, c) HPStDVB; (b, d) HPStDVB\PPy. TEM images of (e, f) cross sections of PPy/HPStDVB composite particles. Magnification 11,000× (a) and 30,000× (b).

After the coating reaction, SEM analysis revealed the formation of PPy only on the HPStDVB surface (Figure 2b), and no free PPy globules were found. TEM analysis proved that PPy was deposited on the surface of HPStDVB microparticles (PPy/HPStDVB composite particles) in the form of a thin, rough layer composed of ∼30 nm PPy globules (Figure 2d) as it was also observed with various PSt latexes coated with a thin PPy layer using different oxidants to produce core–shell PPy/PSt particles.^29,30^ Even if these composite systems exhibited sufficient and comparable PPy loadings, the conductivity of these composite systems was lowered in most cases. They were successfully employed for antistatic, anticorrosion, or biomedical applications.^31,32^ This is not surprising, considering the fact that during oxidative chemical polymerization, hydrophobic pyrrole oligomers are produced and adsorbed on any available surface, in this case, on the HPStDVB microparticles, forming nucleation centers from which subsequent PPy chains are growing. The presence of a PPy film on HPStDVB microparticles was further confirmed by TEM analysis of the cross-section of the PPy/HPStDVB composite particles. Figure 3a,b clearly documents a darker thin layer composed of tiny PPy particles with an average thickness of ∼107 nm on the surface of HPStDVB microparticles.

**Figure 3:**
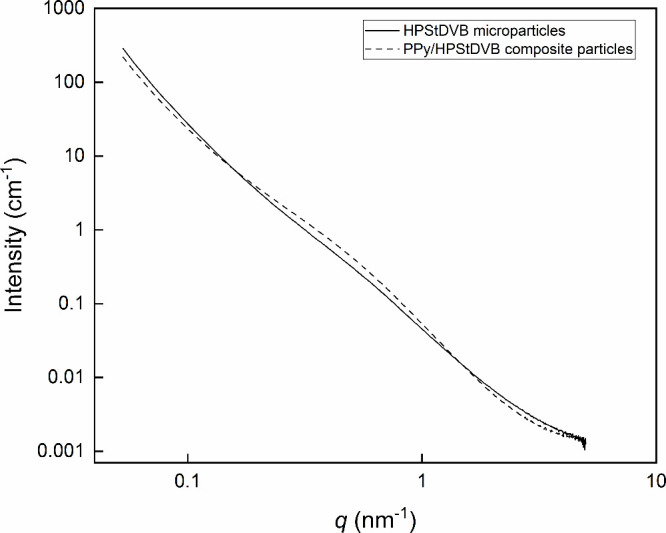
SAXS profiles HPStDVB microparticles (solid line) and PPy/HPStDVB composite particles (dash line).

Besides, SAXS analysis (Figure 3) also confirmed the presence of PPy on the surface of HPStDVB when increased contrast was observed with PPy/HPStDVB composite particles (fitted curve is shown in SI, Figure S3) in comparison with HPStDVB microparticles (fitted curve is shown in SI, Figure S4), documenting an increase in electron density. We also evaluated the average pore size by SAXS analysis, showing that HPStDVB microparticles contained mesopores with the most frequent diameter around 6.3 nm (Figure 3). In PPy/HPStDVB composite particles, a smaller mesopore diameter ∼5 nm was detected. This observation may indicate that the PPy film could partially fill the porous structure of HPStDVB microparticles, leading to a decrease of the average pore diameter. Besides, the arrangement of PPy globules into the form of a thin PPy film on the surface of HPStDVB microparticles could result in the formation of additional porous structure, which is obvious in the TEM image (Figure 2d) of PPy/HPStDVB composite particles.

This hypothesis was also confirmed by physisorption measurements to determine *S*_BET_, total pore volume (TPV), and average pore diameter (APD). From the adsorption isotherms (shown in SI, Figure S5), it was determined that HPStDVB microparticles exhibited values of *S*_BET_ = 247 m^2^/g, TPV = 0.20 cm^3^/g, and APD = 3.30 nm. For PPy/HPStDVB composite particles, the presence of PPy increased both the specific surface area (*S*_BET_ = 303 m^2^/g) and the total pore volume (TPV = 0.27 cm^3^/g), along with a slightly larger average pore diameter (APD = 3.57 nm), which could be attributed to an increase in mesopores.^33^ These results indicate the formation of a finer and more complex porous structure in the PPy/HPStDVB composite particles and further confirm the successful preparation of a porous, conductive composite suitable for electrochemical applications.

### 3.1 NMR Spectroscopy

^13^C CP/MAS NMR spectroscopy was used to investigate the structural features of PPy and HPStDVB microparticles in the final PPy/HPStDVB composites. Experimental ^13^C CP/MAS NMR spectra of the pristine systems and PPy/HPStDVB composite particles are depicted in Figure 4. The ^13^C CP/MAS NMR spectrum (Figure 4a) reveals two resonances at around 114 and 127 ppm (with error ± 5 ppm), assigned to α- and β-carbons of the pyrrole rings, respectively. On the other hand, in the ^13^C CP/MAS NMR spectrum of HPStDVB (Figure 4b), four dominant peaks were detected. Signals at 127 and 146 ppm correspond to quaternary aromatic (=C_Ar_=) carbons and protonated aromatic carbons (=CH_Ar_−), respectively. Other signals in the aliphatic region with chemical shift ca. 40, 30, and 18 ppm were attributed to peak arising from the reduced methylene (−CH_3_) groups, methylene bridges, and aliphatic methine, respectively.^35^ Moreover, due to the presence of strong spinning-side-bands (*ssb) observed for aromatic carbons of HPStDVB microparticles, the central signal reflecting the isotropic chemical shift was determined by conducting ^13^C CP/MAS NMR experiments at two different spinning speeds. While the resonance frequency of the central signal is MAS-independent, the position of spinning-side-bands directly depends on the applied frequency of MAS (see SI, Figure S6).

**Figure 4:**
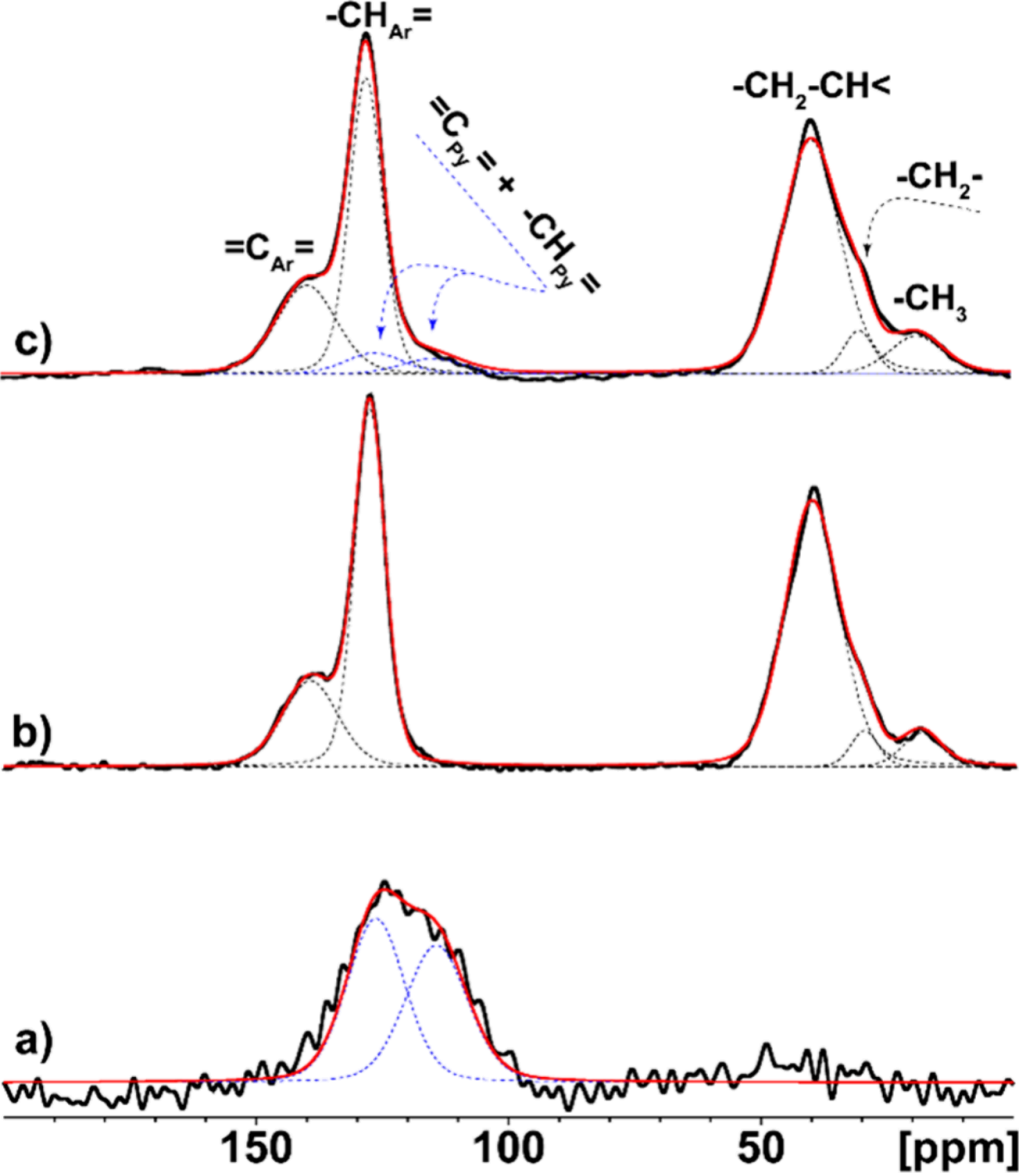
Experimental ^13^C CP/MAS NMR spectra (black solid line), simulations of the individual carbon atoms (dashed lines), and their sum (red solid line) of (a) pristine PPy, (b) HPStDVB (95:5), and (c) PPy/HPStDVB composite particles.

In the case of the final PPy/HPStDVB composite particles, all seven peaks corresponding to both pristine polymers were detected, clearly confirming the formation of PPy in the HPStDVB composite. Additionally, experiments based on the ^13^C CP/MAS NMR technique revealed detectable broadening of all signals in the case of composite material, which, based on the analysis of ^1^H–^13^C PILGRIM/MAS NMR spectra, can be attributed to the reduced segmental dynamics in HPStDVB caused by interactions with PPy chains.

In general, the 2D ^1^H–^13^C PILGRIM spectra allow us to monitor the strength of site-specific ^1^H–^13^C dipolar interactions for each carbon atom and molecular segment spectroscopically resolved in the ^13^C CP/MAS NMR spectra.^27^ While information about isotropic chemical shifts of ^13^C atoms is provided in the direct dimension *F*_2_, the magnitude of ^1^H–^13^C dipolar interactions is stored in the indirect dimension *F*_1_ in the form of dipolar profiles (Figure 5a). The dipolar profiles are then separated for each resolved ^13^C NMR resonance. Then, for a selected dipolar profile, for instance, for an aromatic CH unit, the outer, usually well-resolved doublets reflect one-bond proton-carbon dipolar interactions, while the inner dipolar profile describes medium- and long-range dipolar couplings originating from more distant neighboring protons. Generally, for completely rigid segments and using the PILGRIM experiment with the scaling factor cos(54.7), the splitting of the outer doublets should be ca. 23.5 kHz. Any reduction of the detected splitting then reflects averaging of dipolar interactions induced by segmental dynamics.^27,36^

**Figure 5:**
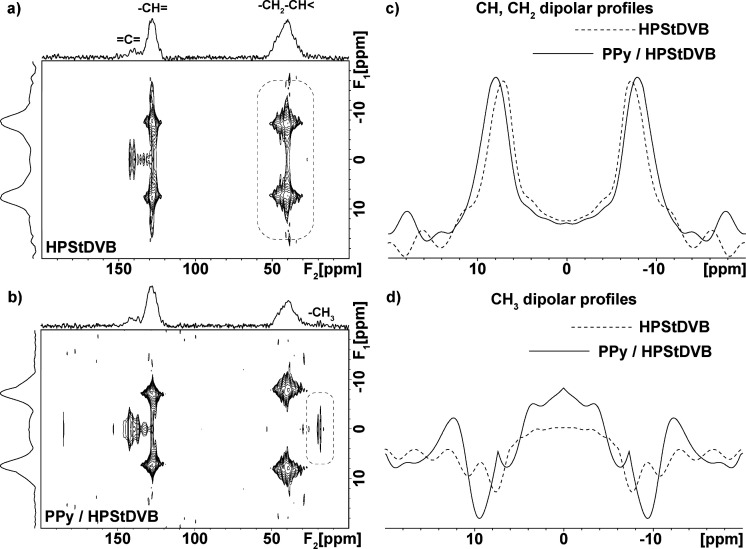
Experimental ^1^H–^13^C PILGRIM NMR spectra of (a) HPStDVB (95:5); (b) PPy/HPStDVB composite; (c) dipolar profiles selected for CH and CH_2_ backbone segments; and (d) methyl groups.

Specifically, for pristine HPStDVB microparticles, the detected outer splitting for aromatic and aliphatic segments falls in the range from 17.5 to 19.2 kHz, which is the value typical for rigid polymer networks with a relatively open structure allowing to execute low-amplitude segmental reorientations (Figure 5a).^37^ In contrast, for pristine PPy, it was impossible to record any dipolar profile. This resulted from the fast *T*_1_ and *T*_2_ spin-relaxation processes induced by the presented polaron(s) on the PPy polymeric chains. Consequently, due to the high conductivity of PPy, the ^1^H–^13^C cross-polarization and dipolar oscillation are nearly completely attenuated.

When analyzing the ^1^H–^13^C PILGRIM spectrum of PPy/HPStDVB composite particles, we see a slight attenuation of the signal intensity of aliphatic segments resonating at ca. 40–45 ppm (Figure 5b), which is accompanied by a slight increase in the corresponding dipolar splitting (Figure 5c). This phenomenon can be explained by interactions of the HPStDVB backbone with PPy chains, inducing a partial loss of ^1^H–^13^C coherence and a reduction of segmental dynamics. In contrast, in the detected ^1^H–^13^C PILGRIM spectrum of the composite system PPy/HPStDVB, we also see an increase in the intensity of dipolar signals of nonprotonated aromatic carbons and mobile methyl groups (Figure 5d). These findings then indicate some type of rearrangement of polymer segments toward a more compact structure, which causes a reduction of the reorientation amplitudes of methyl groups and aromatic substituents of the HPStDVB polymers and allows more efficient long-range transfer of ^1^H polarization to nonprotonated carbon atoms. Therefore, we can suppose that PPy chains, the amount of which is relatively low in the composite (16 wt %; calculated from elemental analysis SI, Table S1), are rather homogeneously dispersed and distributed throughout the polymer matrix.

### 3.2 ATR FTIR Spectroscopy

In the ATR FTIR spectrum of HPStDVB microparticles (Figure 6, line b), absorption bands at 817 and 700 cm^–1^ were attributed to p-substituted benzene rings, which proved the cross-linking at the para position.^34^ Moreover, peaks around 2915 cm^–1^ correspond to the −CH_2_– symmetric and antisymmetric stretching vibrations, confirming the presence of methylene bridges in HPStDVB microparticles after the hyper-cross-linking reaction.^38,39^

**Figure 6:**
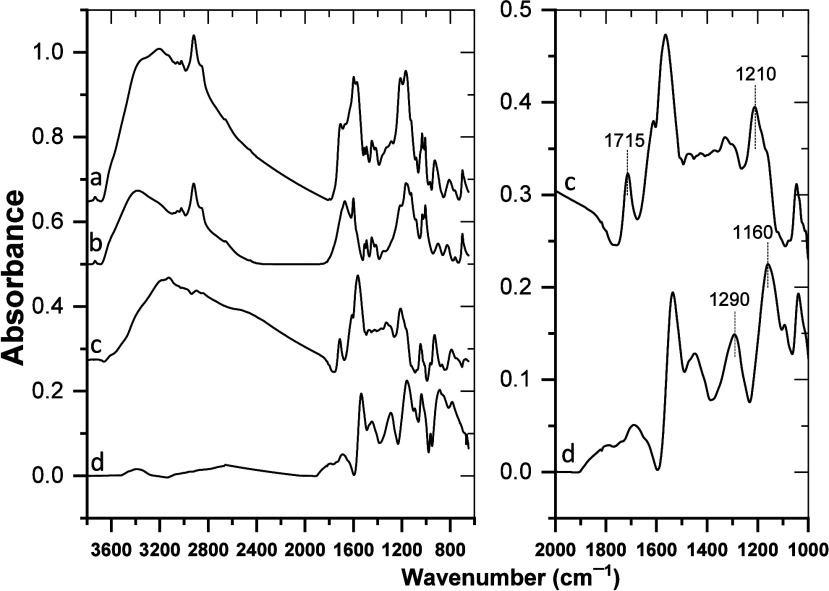
ATR FTIR spectroscopy. Left panel: (a) spectrum of PPy/HPStDVB composite particles, (b) spectrum of HPStDVB microparticles, (c) difference spectrum obtained by subtraction of the HPStDVB microparticles spectrum (line b) from the spectrum of PPy/HPStDVB composite particles (line a), and (d) spectrum of pristine PPy. Right panel: details of spectra (c, d) from the left panel.

The broad band occurring at around 3390 cm^–1^ in the ATR FTIR spectrum of pristine PPy (Figure 6, line d) is attributed to the N–H stretching vibration in pristine PPy, while the C–H stretch would normally be found at 2927 cm^–1^, but the bands in this PPy spectrum have a derivative line shape, and this makes the determination of the precise position of the band maxima more involved. The band at approximately 1540 cm^–1^ is due to the C=C stretching vibration and/or due to the C–C in-ring stretch. The peak observed at around 1450 cm^–1^ is attributed to the ring-breathing mode combined with C=C/C–C and/or C–N stretching modes. The band located at approximately 1290 cm^–1^ is assigned to C–N in-plane bending and C–H in-plane bending, while the band at around 1160 cm^–1^ represents C–N stretching.

In addition, Arjomandi et al. pointed out that the infrared spectra of pristine PPy prepared under different conditions by various laboratories were not identical due to the different extents of charge delocalization.^40^ However, these authors also stated that despite the differences in charge delocalization, the prominent vibrational bands of PPy are at approximately the same position.

All of the characteristic bands of HPStDVB microparticles were also found in the spectrum for the PPy/HPStDVB composite particles (Figure 6, line a). Moreover, this spectrum of PPy/HPStDVB composite particles contains the band for the C=C stretching vibration and/or due to the C–C in-ring stretch and the band for the C–N in-plane bending and C–H in-plane bending, which are at approximately the same positions as in the spectrum of pristine PPy (Figure 6, line d), at 1540 cm^–1^ and 1290 cm^–1^, respectively. It is not possible to make these comparisons more quantitative or precise, since the derivative line shape of the peaks in the spectrum of PPy/HPStDVB composite particles (Figure 6, line a) is less pronounced or none, as opposed to the pristine PPy, due to the low amount of PPy in the composites and the different properties of the composite particles as opposed to pristine PPy.

Furthermore, the band at around 1210 cm^–1^ in the difference PPy spectrum (Figure 6, line c) dominates in the region between ∼1300 cm^–1^ and ∼1100 cm^–1^ and corresponds to radical cations (polarons), while the band at around 1160 cm^–1^ dominates the corresponding region in pristine PPy and is associated with dications (bipolarons).^41^ The PPy in PPy/HPStDVB composite particles thus appears only slightly oxidized, while the pristine PPy is in the more oxidized form. It is therefore puzzling why a band at around 1715 cm^–1^ is also present in the difference PPy spectrum, since this band is associated with the C=O stretching vibration of carbonyl groups forming in overoxidized (and not in slightly oxidized) PPy, and the newly formed carbonyl groups would cause interruptions in the conjugation of PPy, which would result in a loss of electroactive capacity of PPy. There is no carbonyl stretching band present in the pristine PPy spectrum (Figure 6, line d) or in the spectrum of HPStDVB microparticles (Figure 6, line b).

The difference PPy spectrum (Figure 6, line c), which was obtained by subtraction of the HPStDVB microparticles spectrum (Figure 6, line b) from the spectrum of PPy/HPStDVB composite particles (Figure 6, line a), shows that the region from ∼3500 to ∼2,000 cm^–1^, where normally N–H stretch and C–H stretch of PPy would be found, comprises an asymmetrical and extremely broad band. The asymmetry toward lower wavenumbers and the extreme width of this band are most likely caused by scattering of the infrared radiation by the microparticles, since this difference is not a spectrum of pristine bulk PPy but a spectrum of PPy that is distributed over the pores of HPStDVB microparticles (as suggested by NMR spectroscopy above) with a diameter comparable to the wavelength of the used infrared waves with the given spectral range. The scattering effects manifest themselves indeed in the spectrum of HPStDVB microparticles (Figure 6, line b) and in the spectrum of PPy/HPStDVB composite particles (Figure 6, line a). In the latter spectrum, the scattering extends over a wider region (∼3500 to ∼2000 cm^–1^) than in the former spectrum (∼3500 to ∼2500 cm^–1^), indicating a wider distribution and greater diameters of the PPy/HPStDVB composite particles than those found in the original HPStDVB microparticles. This agrees with the formation of thin, rough PPy layers on the surface of HPStDVB particles observed by TEM above.

In addition to the scattering effects, it was expected that under the laboratory conditions applied, water adsorption took place throughout the HPStDVB microparticles. The O–H stretching vibration can extend over the spectral region between ∼3700 and ∼3000 cm^–1^ and thus overlaps with the scattering effects due to the microparticles. The shift from ∼3400 to ∼3250 cm^–1^ in the position of the observed band maximum, when going from the original to the PPy-covered form of microparticles, indicates that the water molecules adsorbed throughout the PPy-covered form of microparticles are more strongly bound than the water molecules adsorbed throughout the original HPStDVB microparticles.

The fact that pyrrole polymerizes into PPy on the surfaces of poly­(styrene-*co*-divinylbenzene) pores in the presence of immobilized water enables the water molecules to cause interruptions in the conjugation of PPy and to take part in the formation of carbonyl groups, even though electrochemically there is no reason for PPy to be overoxidized and to have the conjugation disturbed with built-in carbonyl groups as a result of overoxidation. The PPy covering the microparticles is, according to the presence of the band at around 1210 cm^–1^, only slightly oxidized. Overall the PPy structure and electrochemical properties of the PPy/HPStDVB composite are more stable due to less dynamic functional groups, as also suggested by NMR spectroscopy above.

### 3.3 Electrochemical Properties

#### 3.3.1 Three-Electrode System

CV, EIS, and GCD measurements were performed for the PPy/HPStDVB electrode in a three-electrode system. The CV was performed over a range of 0 to 0.75 V with scan rates ranging from 10 to 200 mV/s. The resulting CV curve exhibited a nearly rectangular shape with good cyclic reversibility for all scan rates, indicating a supercapacitor-like electrochemical response of the PPy/HPStDVB electrode. Furthermore, *C*_A_ was calculated using the relation between current and scan rate (using eq 3), as shown in Figure 7a. The highest *C*_A_ of 4.1 mF/cm^2^ was obtained at a scan rate of 10 mV/s. Furthermore, as the scan rate increased, *C*_A_ showed a decreasing trend (Figure 7a). This is due to the fact that at lower scan rates, the electrolyte can penetrate into the pores more thoroughly and make better contact with the internal surface of the PPy/HPStDVB electrode, hence resulting in a higher *C*_A_.

**Figure 7:**
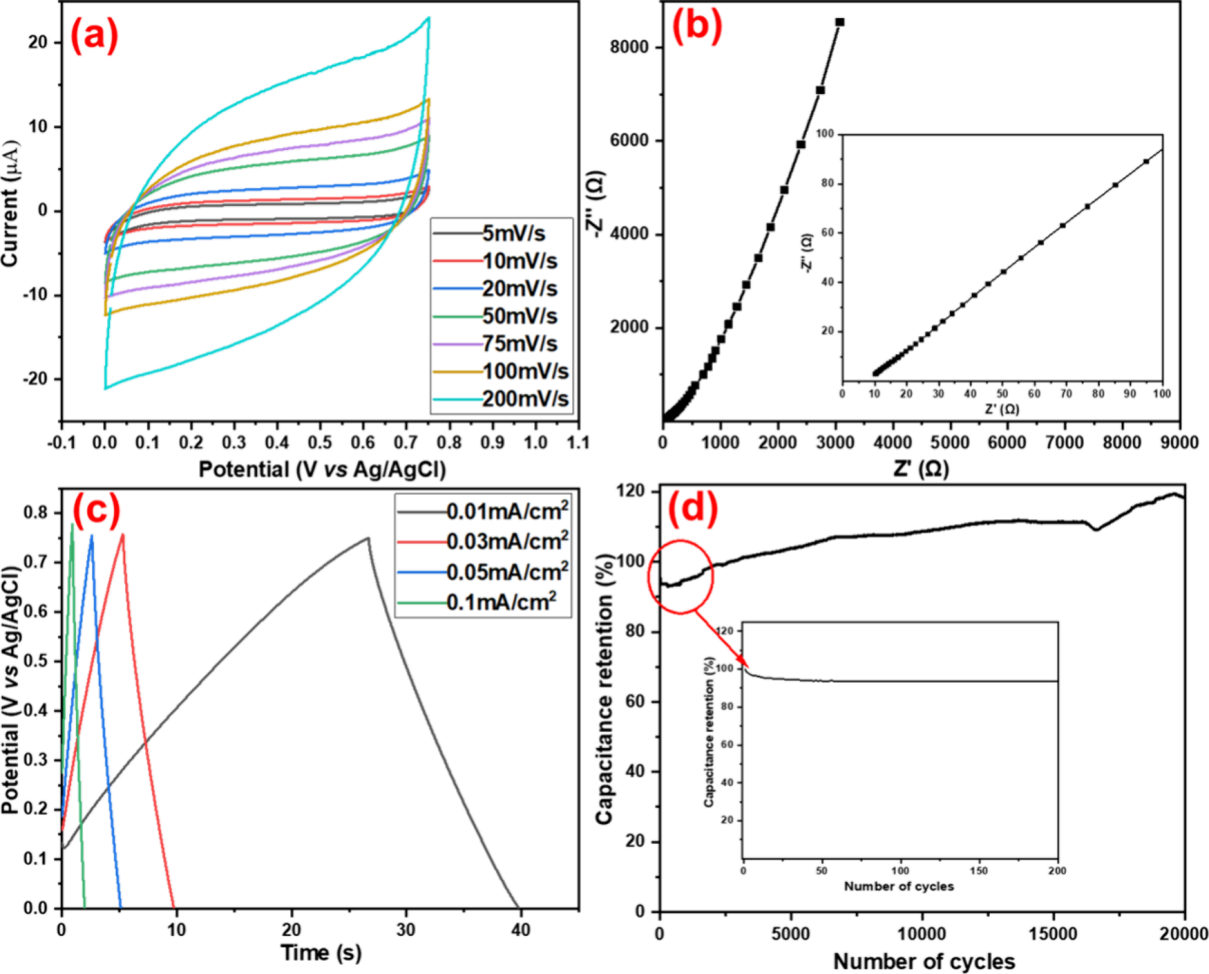
Electrochemical studies of the three-electrode system in 1 M HCl; (a) CV from 5 mV/s to 200 mV/s; (b) EIS from 0.1 Hz to 10^5^ Hz with the amplitude of 5 mV; (c) GCD 0.01 to 0.1 mA/cm^2^; and (d) cyclic stability up to 20,000 cycles of the PPy/HPStDVB-coated electrode.

The Nyquist plot illustrates the EIS spectrum of the PPy/HPStDVB electrode used to investigate its capacitive and resistance properties within the frequency range of 0.1 Hz to 10^5^ Hz with an amplitude of 5 mV (Figure 7b). The plot shows two major regions. The high-frequency region indicates that the equivalent series resistance (ESR) is 10.24 Ω/cm^2^. It generally originated due to the solution resistance and contact resistance between the PPy/HPStDVB coating and glassy carbon. The other part, showing a steep rise in behavior parallel to the imaginary part of the impedance in the low-frequency region, indicates the capacitive behavior of the PPy/HPStDVB electrode. Furthermore, *C*_A_ was calculated from this spectrum using eq 5. The *C*_A_ of the PPy/HPStDVB electrode is 2.62 mF/cm^2^.

Furthermore, GCD was also performed on the PPy/HPStDVB electrode to investigate its charging and discharging responses. The GCD showed a nearly triangular shape of curve, indicating the capacitive behavior of the PPy/HPStDVB electrode (Figure 7c). The PPy/HPStDVB electrode showed almost consistent *C*_A_ at all applied current densities, which indicates its better electrochemical response. The highest *C*_A_ of 2.83 mF/cm^2^ was recorded at 0.05 mA/cm^2^. Also, the PPy/HPStDVB electrode exhibited excellent stability up to 20,000 cycles at a scan rate of 100 mV/s (Figure 7d). Despite the excellent electrochemical performance of PPy-based electrodes, they are subjected to volume expansion and structural damage during long-term cyclic stability due to the doping and undoping processes.^42–45^ However, in the present case, HPStDVB provides better structural support to PPy in PPy/HPStDVB, which restricts its volume expansion and any other structural changes; therefore, the PPy/HPStDVB electrode showed excellent cyclic stability for up to 20,000 cycles (Figure 7d and see SI, Figure S7), which is higher than our previously reported PPy (90% after 5000 cycles at 100 mV/s).^46^ Figure 7d shows an initial slight decrease in capacitance retention, which may be due to the lower wettability of the electrode. Also, sometimes, the electrode takes time to optimize the full use of its surface. After achieving sufficient wettability, it retains its original cyclic stability for up to 20,000 cycles.

#### 3.3.2 Cell Study

After conducting a three-electrode electrochemical study of the PPy/HPStDVB electrode, a cell was fabricated using a PPy/HPStDVB-coated carbon cloth electrode, as shown in Figure 1. Furthermore, the cell was tested for CV, EIS, GCD, and cyclic stability. The CV curves were taken at different scan rates ranging from 1 to 500 mV/s with a fixed potential window of 0 to 0.75 V. The cyclic behavior of the cell was found to be similar to that of the three-electrode system. The CV curves show excellent reversibility over the selected potential window, exhibiting a rectangular shape. This indicates the capacitive behavior of the cell as well as the formation of electrical double layers at the interface of the electrode and the electrolyte. At lower scan rates, the CV curves are more rectangular in shape (1–75 mV/s). However, at higher scan rates, the CV curves show slight tilting (100 and 200 mV/s) (Figure 8a,b). Previous studies have observed similar kinds of CV behaviors in PPy composites.^47^ Such behavior at lower scan rates suggests the uninterrupted transport of electrolyte ions into the pores of PPy/HPStDVB with the formation of the electrical double layer. On the other hand, at higher scan rates, the tilting of CV curves indicates substantial internal resistance with lower migration of electrolyte inside the pores, leading to lowering in *C*_g_.^47^ Therefore, with this cell, the highest *C*_g_ of 12.21 F/g was obtained at a scan rate of 1 mV/s using eq 4. After this, the *C*_g_ decreased gradually with increasing scan rates (Figure 9a,b). The Nyquist plot of the EIS spectrum was recorded under the same parameters which were used in the three-electrode system. This plot showed a low ESR of 2.59 Ω/cm^2^ in the high-frequency region and capacitive behavior in the low-frequency region, which is parallel to the imaginary part of the impedance. These features of the Nyquist plot indicate the supercapacitive behavior of the cell. Furthermore, a *C*_g_ of 6.66 F/g was calculated from the low-frequency region of this plot using eq 6.

**Figure 8:**
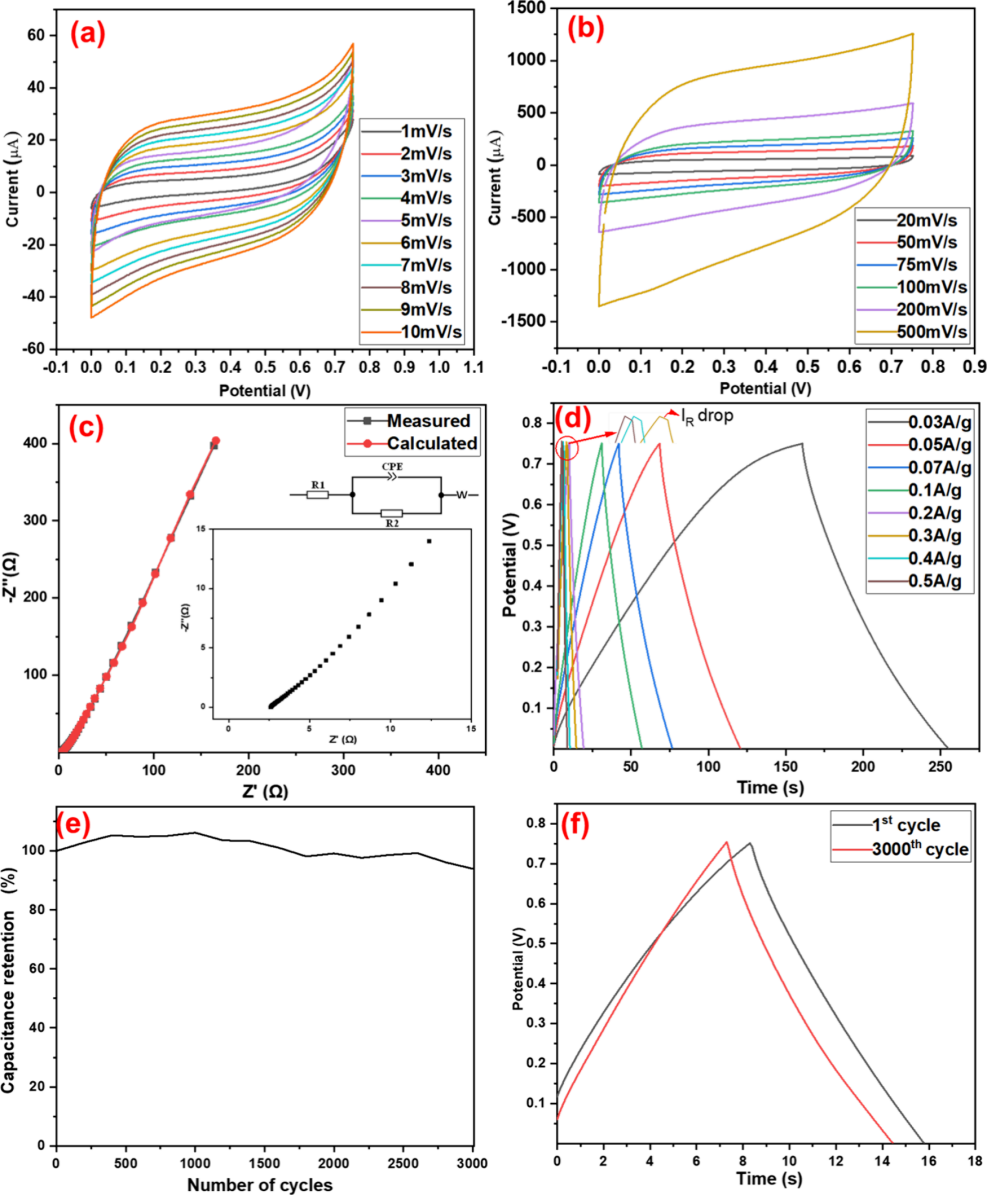
Electrochemical studies of the PPy/HPStDVB-based cell in 1 M HCl; (a) CV from 1 to 10 mV/s; (b) CV from 20 to 500 mV/s; (c) EIS from 0.1 Hz to 10^5^ Hz with the amplitude of 5 mV (insets: circuit diagram and EIS in the high-frequency region); (d) GCD from 0.03 to 0.5 A/g; (e) cyclic stability of cell up to 3000 charging discharging cycles; and (f) first and last cycles of GCD.

**Figure 9:**
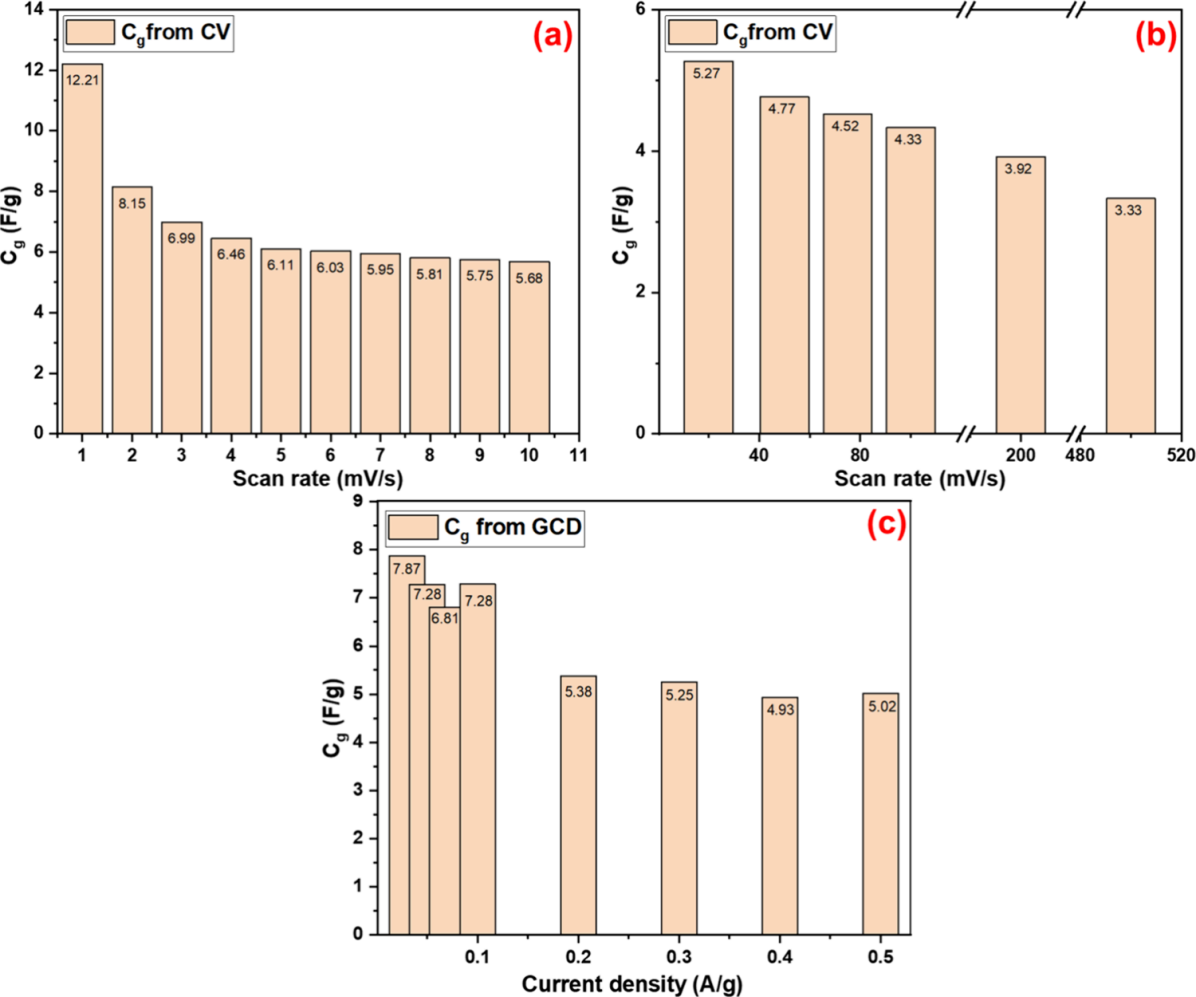
(a) and (b) *C*_g_ with scan rates from CV (c) *C*_g_ with scan rates from GCD for the cell.

The GCD was performed for the PPy/HPStDVB-based cell at different current densities from 0.03 to 0.5 A/g within the window potential of 0 to 0.75 V (Figure 8d). The GCD curve shows nearly a triangular, supercapacitor-type charging discharging behavior. As the current density increased (from 0.3 to 0.5 A/g), distortion in the triangular shaped occurred along with an increase in the *I*_R_ drop or voltage drop, as shown in Figure 8d. This drop is generally originated due to the resistance in the cell, which includes the resistance of the electrode material (in PPy/HPStDVB-based cell), the electrolyte, and the interface of the electrode and the electrolyte. This resistance is usually higher at higher current densities.^48–50^ The highest *C*_g_ value of 7.87 F/g was calculated at a current density of 0.03 A/g (Figure 9b) using eq 8.

Furthermore, *C*_g_ was also calculated at other current densities, which can be seen in Figure 9c, revealing a decrease in *C*_g_ values as the current density increased. This phenomenon occurs because during the GCD measurement, the amount of charge stored at constant current density depends on time. At higher current densities, due to the faster process, the time available for charge accumulation is reduced, leading to lower *C*_g_ values.^51^ Furthermore, cyclic stability of the cell was also performed up to 3000 charging discharging cycles at 0.3 A/g, as shown in Figure 8f. The cyclic stability test of the cell showed 93% retention of its initial capacitance. The energy density (*E*_d_) and power density (*P*_d_) were calculated for the PPy/HPStDVB-based cell using eqs 9 and 10, respectively. The cell showed an *E*_d_ of 0.61 Wh/kg and a *P*_d_ of 22.63 W/kg. According to Table 1, the electrochemical stability of PPy/HPStDVB was found to be better or comparable to previously reported materials.

**Table 1:** Comparison of Electrochemical Performance of Recently Reported PPy or Conducting-Polymer Composite Electrode^a^

electrode material	electrolyte	capacitance	current density/scan rate	cyclic stability	system	ref.
PPy-stabilized polypeptide	PVA/PBS hydrogel	4 F/g	0.1 A/g	94.8% after 5000 cycle	Cell	^52^
PyHCP-800/PANI (PyHCP-800 after carbonization)	6 M KOH	15.83 F/g	0.1 A/g	90.67% after 2000 cycle	Cell	^53^
nano-PANI/HCS (HCS after carbonization)	1 M H_2_SO_4_	435 F/g	0.5 A/g	60% after 2000 cycles	3-Ele	^54^
hyper-cross-linked DVB-co-VBC/GNP	0.5 M H_2_SO_4_	60.4 F/g	10 mV/s	97% after 10000 cycles	3-Ele	^55^
DVB:VBC-800 (after carbonization)	3 M KOH	145 F/g	1 A/g	94% after 5000 cycles	Cell	^56^
PPy at room temperature	0.2 M HCl	20 F/g	10 mV/s	90% after 5000 cycles	3-ele	^46^
PPy/HPStDVB	1MHCl	4.1 mF/cm^2^	10 mV/s	no capacitance loss was observed after 20000 cycles	3-Ele	this work
		12.21 F/g	1 mV/s	93% after 3000 cycles	Cell	this work

a PyHCP: hypercross-linked polymer of pyrene; PANI: polyaniline; HCS: hollow carbon spheres (prepared from poly­(styrene-co-divinylbenzene-co-methylacrylic acid)); GNP: graphite nanoplatelets; DVB-co-VBC: poly­(divinylbenzene-co-vinylbenzyl chloride).

PPy and PPy-based materials are known for their redox or faradic behavior, which is controlled by ion diffusion processes.^57,58^ Generally, in SCs, the electrochemical response of these materials is largely capacitive in appearance, with redox or faradic essence.^59,60^ Therefore, the capacitive contributions to the total *C*_g_ of the PPy/HPStDVB-based cell were calculated. For this, CV was run at low scan rates (6–10 mV/s) (Figure 10a,b). The value was calculated using the following equation:^61^

$$i=i_{\mathrm{capacitive}}+i_{\mathrm{diffusion}}=ak^{b}$$ 11

where *i* is the total current, *i*_capacitive_ is the capacitive current, *i*_diffusion_ is the diffusion current, *k* is the scan rate, and *a* and *b* are adjustable parameters.

**Figure 10:**
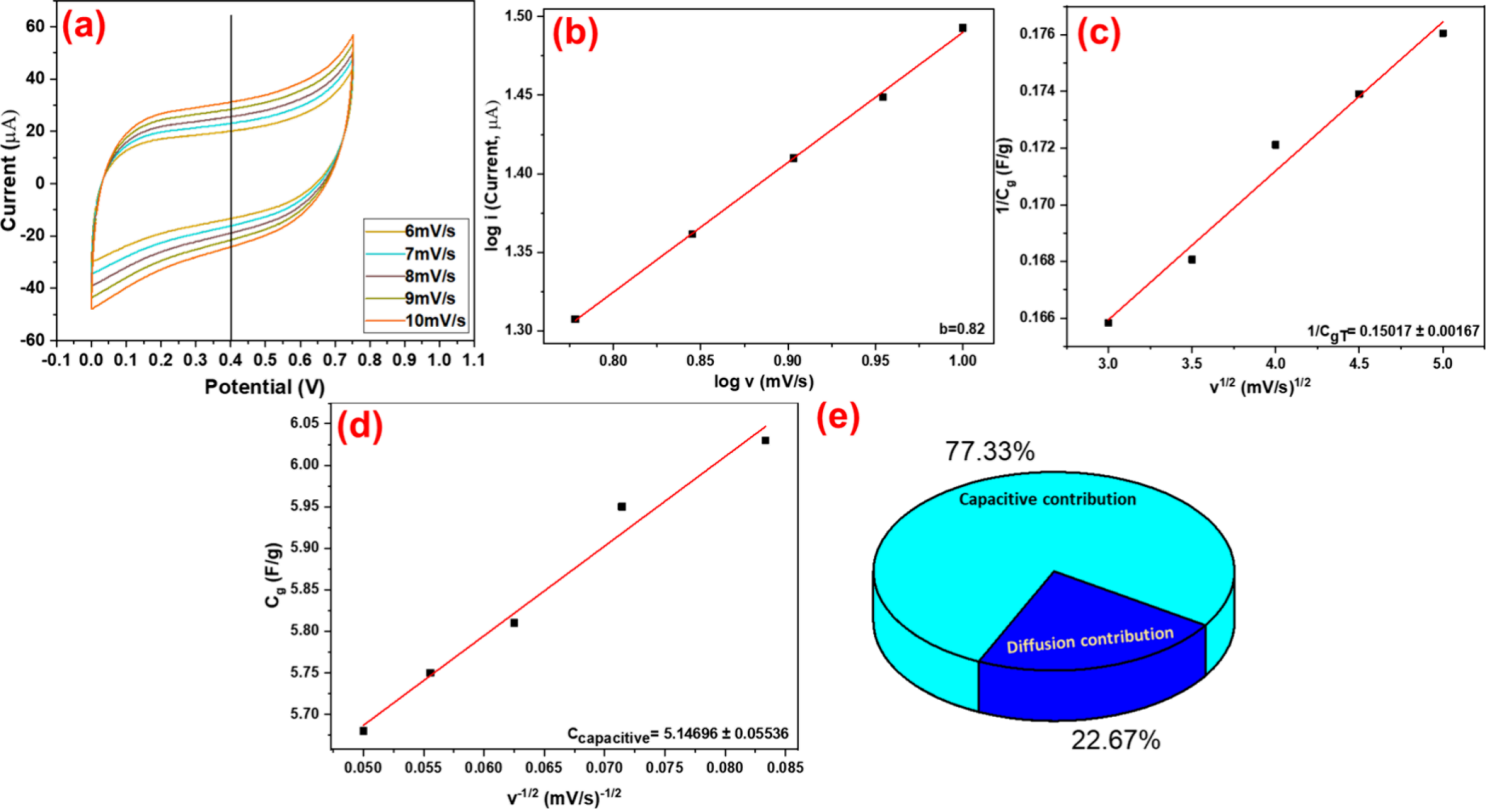
(a) CV curve at different scan rates from 6 to 10 mV/s; (b) *b* value from log *i* vs log *v*; (c) plot of reciprocal of $\frac{1}{C_{g}}$ vs square root of scan rate (*v* ^1/2^); (d) plot of *C*_g_ vs reciprocal of square root of scan rate ( $\frac{1}{v^{1/2}}$ ); and (e) capacitive contribution.

The log transformation of this equation turns into log *i* = *b* log *k* + log *a,* so that a simple linear equation can be fitted to calculate the *b* value from the graph between log *i* vs log *k* (Figure 10b). Generally, the *b* value defines the contribution of capacitance in the cell: if *b* = *1*, it is capacitive; if *b* = *0.5*, it is diffusion-controlled process.^62^ In the PPy/HPStDVB-based cell, the *b* value was 0.82, which is close to 1, indicating a more capacitive contribution than diffusion contribution.

Furthermore, for the confirmation of more capacitive contribution and contribution percentage of capacitive and diffusion-controlled processes of the PPy/HPStDVB-based cell, the Trasatti method was employed using the following equations.^63^ Initially, the maximum or total capacitance, maximum capacitive, and diffusion capacitances were calculated as follows:

$$\frac{1}{C_{g}}=\mathrm{cons}\tan t\cdot v^{1/2}+\frac{1}{C_{\mathrm{rg}}}(\mathrm{maximum}\;\mathrm{capacitance})$$ 12

where *C*_g_ is the gravimetric capacitance, *v* is the scan rate, and *C*_Tg_ is the total or maximum capacitance (capacitive and diffusion). *C*_Tg_ equals the reciprocal of the y-intercept of 1/*C*_g_ vs *v* ^*1/2*^ (Figure 10c). The calculated value of 1/*C*_g_ for the cell is 0.15017.

Furthermore, the maximum capacitive and diffusion capacitance contributions were calculated using the following equation:

$$C_{g}=\mathrm{cons}\tan t\cdot \frac{1}{v^{1/2}}+C_{\mathrm{capacitive}}$$ 13

Using this equation, a plot of *C*_g_ vs the reciprocal of the square root of scan rates ( $\frac{1}{v^{1/2}}$ ) was drawn, as shown in Figure 10d. From this plot, the maximum capacitive contribution was calculated via fitting a linear equation. The value of *C*_capacitive_ is 5.1496. Subsequently, subtracting *C*_capacitive_ from C_Tg_ gives the maximum diffusion contribution. The capacitance contribution for both capacitive and diffusion can be calculated using the following equations:

$$C_{\mathrm{capacitive}}\;\%=C_{\mathrm{EDL}}/C_{\mathrm{Tg}}\times 100\%$$ 14

$$C_{\mathrm{diffusion}}\;\%=C_{\mathrm{diffusion}}/C_{\mathrm{Tg}}\times 100\%$$ 15

where *c*_capacitive_% and *c*_diffusion_ % are the capacitance percentages of capacitive and diffusion, respectively.

Using the above equations, the capacitance contribution of the PPy/HPStDVB-based cell is 77.33% of capacitive and 22.67% of diffusion contributions (Figure 10e), which clearly supports the *b* value as well as the behavior of the CV graph.

## 4 Conclusions

In summary, our study demonstrates the fabrication of composite PPy/HPStDVB microparticles with excellent cyclic stability (demonstrated up to 20,000 cycles—the results indicate stability for even more cycles) and supercapacitive behavior based on 77.33% of capacitive and 22.67% of diffusion contributions. This success is attributed to the combination of the unique properties of both materials, PPy and HPStDVB. At first, 1.80 μm PStDVB microparticles were prepared by dispersion polymerization and chloromethylated and hyper-cross-linked, resulting in HPStDVB microparticles with a reduced diameter of 1.64 μm due to the formation of a more cross-linked internal structure. Then, pyrrole was polymerized on the surface of the HPStDVB microparticles. SEM, TEM, and SAXS analyses showed that PPy formed a shell composed of 30 nm globules around individual HPStDVB microparticles. This developed composite supercapacitive material was analyzed by using ssNMR and ATR FTIR spectroscopies. Both methods confirmed the formation of the PPy shell on the HPStDVB microparticles. In addition, they explained the more stable electrochemical properties and better cycle life of PPy on HPStDVB due to less dynamic functional groups and the reinforcement of PPy by the HPStDVB microparticles, which actually served as scaffolds.

## Supplementary Material



## Notes

SEM microphotograph of PS particles prepared by dispersion polymerization in EtOH/water, initiated with 4 wt % AIBN, and stabilized with 1.7 wt % PVP, TEM image of cross-section of HPS particle, SAXS profile of HPStDVB microparticle and PPy/HPStDVB, adsorption isotherms of HPStDVB (a) and PPy/HPStDVB particles, experimental ^13^C CP/MAS NMR spectra of HPStDVB conducted at (a) 17 kHz and (b) 12 kHz spinning speed of sample, and cyclic stability: first and last cycle of PPy/HPStDVB-coated electrode and elemental analysis. (PDF)
