# Supplementary material for: Ultrastable Polypyrrole Stabilized by Hyper-Cross-Linked Poly(styrene-co-divinylbenzene) for Long-Cycle Supercapacitor Applications
Source: ACS Appl Polym Mater. 2025 Nov 4;7(21):15026–40. doi: 10.1021/acsapm.5c03208 (PMC12624522; doi:10.1021/acsapm.5c03208)
Supplement: Supplementary file 1 [file ap5c03208_si_001.pdf]

Supporting Information

Ultrastable Polypyrrole Stabilized by Hypercrosslinked Poly(styrene-*co*-divinylbenzene) for Long-Cycle Supercapacitor Applications

Petr Šálek[a,*], Manoj Karakoti[a], Sonal Gupta[a], Libor Kobera[a], Adriana Šturcová[a], Miloš Steinhart[a], Jiří Brus[a], Islam M. Minisy[a], Stefan Breitenbach[b], Christoph Unterweger[b], Jiřina Hromádková[a], Patrycja Bober[a,*]

[a]*Institute of Macromolecular Chemistry, Czech Academy of Sciences, 162 06 Prague, Czech Republic,*

[b]*Wood K plus – Kompetenzzentrum Holz GmbH, 4040 Linz, Austria*

[*]*Corresponding author. E-mail: salek@imc.cas.cz, bober@imc.cas.cz*

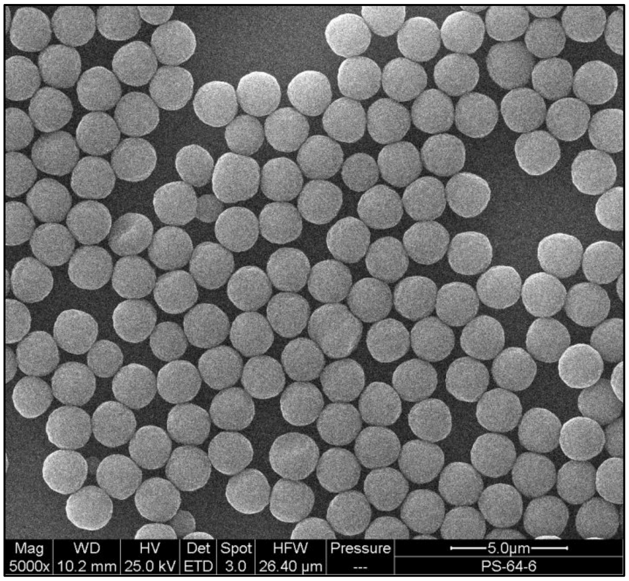

Fig. S1. SEM microphotograph of PS particles prepared by dispersion polymerization in EtOH/water, initiated with 4 wt% AIBN, and stabilized with 1.7 wt% PVP.

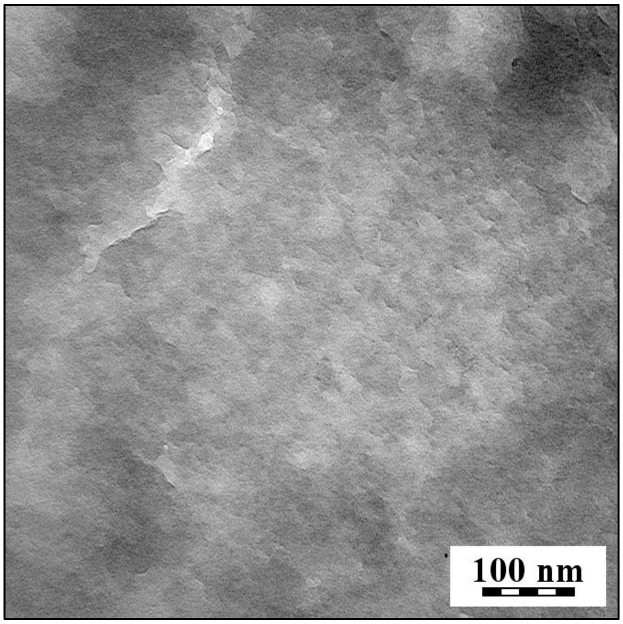

Fig. S2. TEM image of cross-section of HPS particle.

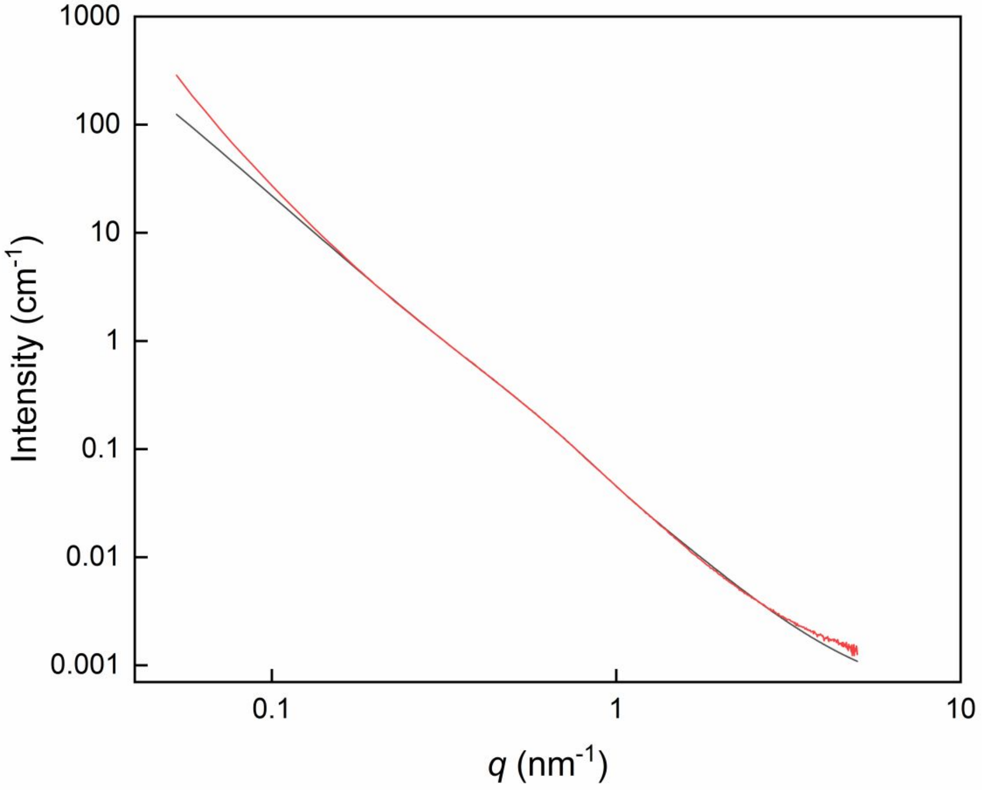

Fig. S3. SAXS profile of HPStDVB microparticle and approximation according to method (red line).

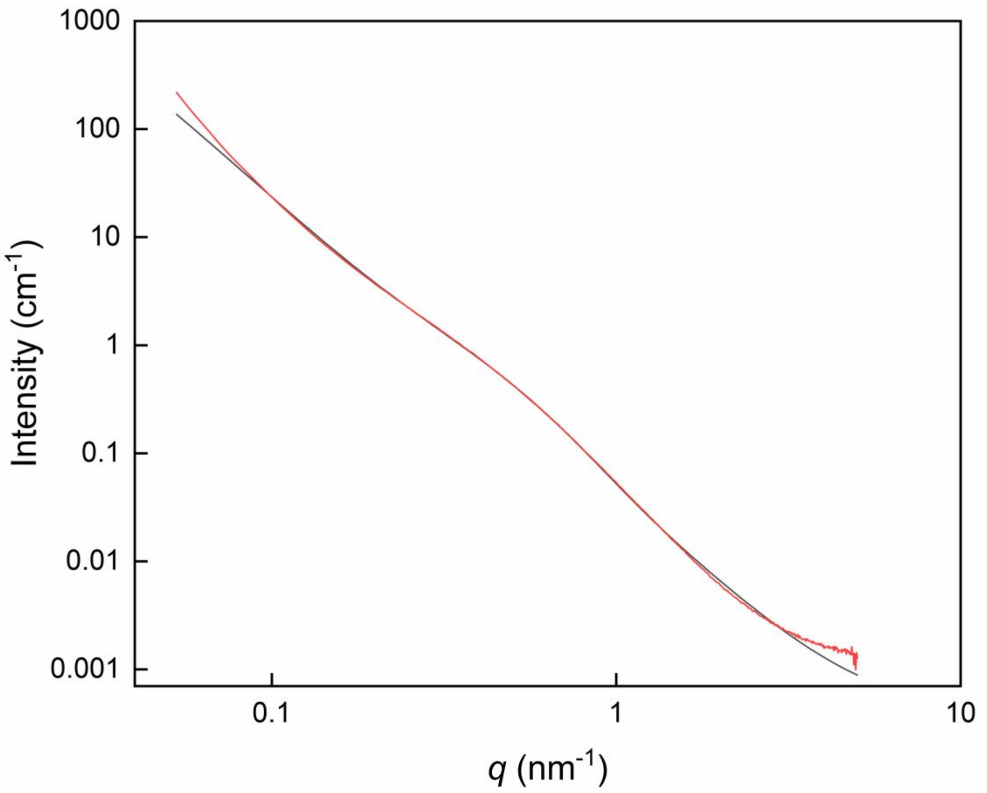

Fig. S4. SAXS profile of PPy/HPStDVB composite particles and approximation according to method (red line).

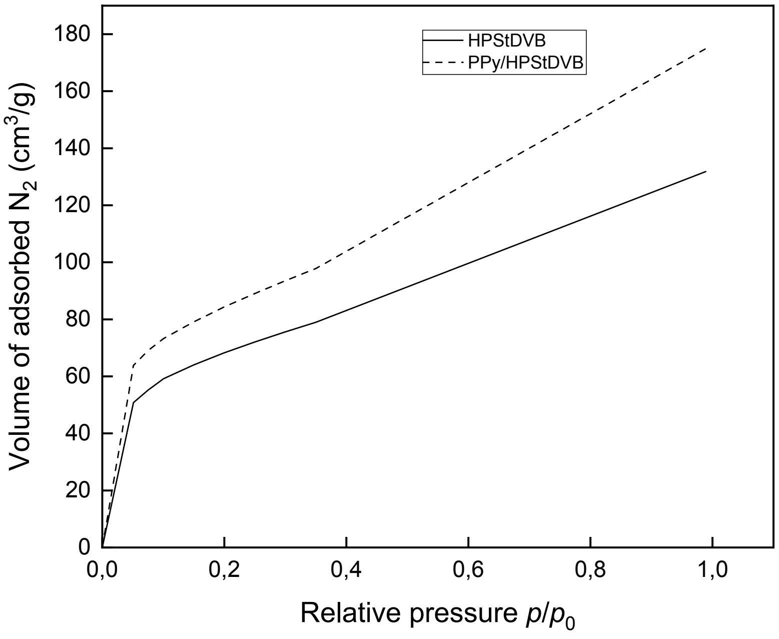

Fig. S5. Adsorption isotherms of HPStDVB (a) and PPy/HPStDVB particles.

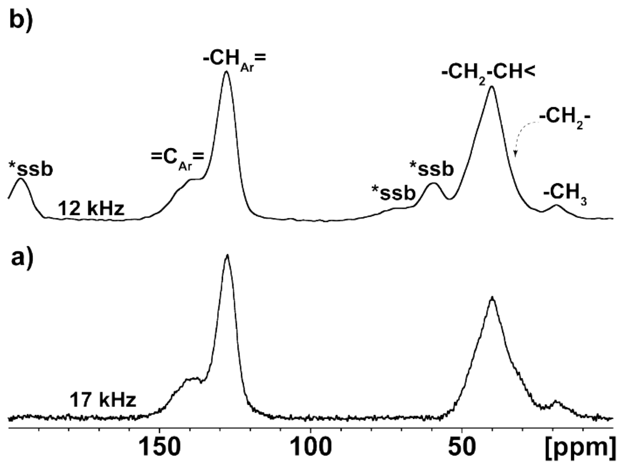

Fig. S6. Experimental ^{13}C CP/MAS NMR spectra of HPStDVB conducted at (a) 17 kHz and (b) 12 kHz spinning speed of sample. The asterisks (*) denote spinning sidebands.

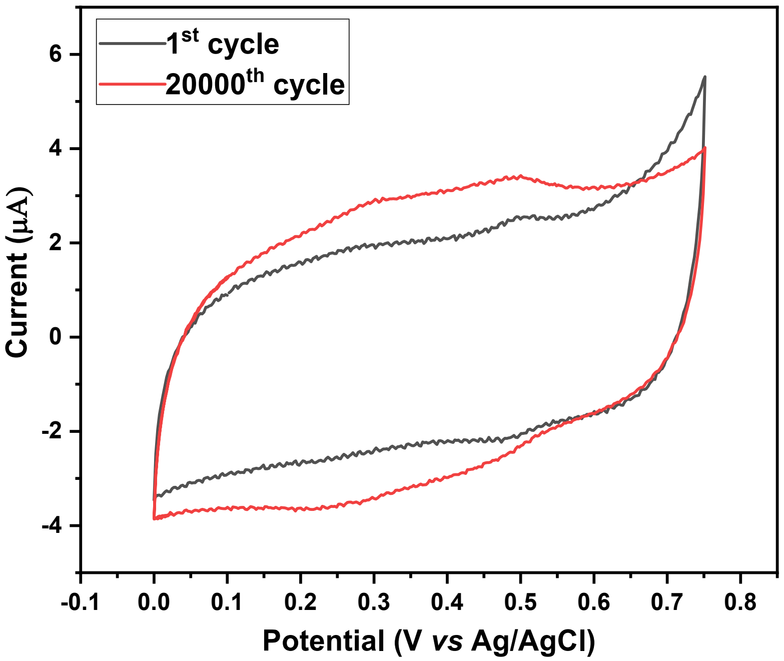

Fig. S7. Cyclic stability: first and last cycle of PPy/HPStDVB coated electrode.

Table S1. Elemental analysis

	**Element**				
**Sample**	**C (wt%)**	**H (wt%)**	**N (wt%)**	**Cl (wt%)**	**Fe (wt%)**
**HPStDVB**	87.05	7.42	0.45	3.93	0.37
**PPy/HPStDVB**	81.46	6.66	3.86	6.61	0.09
